# Bioinspired gradient scaffolds for osteochondral tissue engineering

**DOI:** 10.1002/EXP.20210043

**Published:** 2023-07-12

**Authors:** Yachen Peng, Yaling Zhuang, Yang Liu, Hanxiang Le, Di Li, Mingran Zhang, Kai Liu, Yanbo Zhang, Jianlin Zuo, Jianxun Ding

**Affiliations:** ^1^ Department of Orthopedics China‐Japan Union Hospital of Jilin University Changchun P. R. China; ^2^ Key Laboratory of Polymer Ecomaterials, Changchun Institute of Applied Chemistry Chinese Academy of Sciences Changchun P. R. China; ^3^ School of Applied Chemistry and Engineering University of Science and Technology of China Hefei P. R. China; ^4^ Jilin Biomedical Polymers Engineering Laboratory, Changchun Institute of Applied Chemistry Chinese Academy of Sciences Changchun P. R. China; ^5^ Institute of Bioengineering École Polytechnique Fédérale de Lausanne (EPFL) Lausanne Switzerland

**Keywords:** component, gradient scaffold, osteochondral repair, osteochondrogenesis‐inducing factor, pore, tissue engineering

## Abstract

Repairing articular osteochondral defects present considerable challenges in self‐repair due to the complex tissue structure and low proliferation of chondrocytes. Conventional clinical therapies have not shown significant efficacy, including microfracture, autologous/allograft osteochondral transplantation, and cell‐based techniques. Therefore, tissue engineering has been widely explored in repairing osteochondral defects by leveraging the natural regenerative potential of biomaterials to control cell functions. However, osteochondral tissue is a gradient structure with a smooth transition from the cartilage to subchondral bone, involving changes in chondrocyte morphologies and phenotypes, extracellular matrix components, collagen type and orientation, and cytokines. Bioinspired scaffolds have been developed by simulating gradient characteristics in heterogeneous tissues, such as the pores, components, and osteochondrogenesis‐inducing factors, to satisfy the anisotropic features of osteochondral matrices. Bioinspired gradient scaffolds repair osteochondral defects by altering the microenvironments of cell growth to induce osteochondrogenesis and promote the formation of osteochondral interfaces compared with homogeneous scaffolds. This review outlines the meaningful strategies for repairing osteochondral defects by tissue engineering based on gradient scaffolds and predicts the pros and cons of prospective translation into clinical practice.

## Graphical abstract

The difficulty in repairing osteochondral injuries is mainly the complex gradient properties of natural osteochondral structures. This review discusses the development of various osteochondral tissue engineering scaffolds with gradient properties, including pore gradients, composition gradients, and inducing factor gradients, discusses the future research focus of gradient scaffolds and the trend of osteochondral tissue engineering, and provides ideas for their potential clinical application.

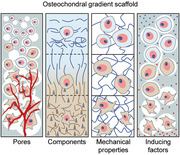

## 1 INTRODUCTION

The osteochondral defect is often secondary to traumatic injuries to the knee, such as a ligamentous and meniscal lesion. The traumatic force is transmitted to subchondral bone through cartilage, leading to osteochondral damage.^[^ ^1^ ^]^ Pain, joint dysfunction, and mobility problems caused by osteochondral injuries are physical and psychological burdens for patients and a severe socioeconomic burden.^[^ ^2^ ^]^

Conventional clinical treatments for osteochondral lesions, including microfracture, autologous/allograft osteochondral transplantation, and cell‐based techniques, act on relieving pain to some extent in short order. Nevertheless, long‐term results are unsatisfactory, and the treatments may lead to adverse complications.^[^ ^3^ ^]^ Specifically, after microfracture, the cartilage at the position will regenerate through fibrocartilage containing large amounts of type I collagen (Col I) with low mechanical properties.^[^ ^4^ ^]^ In addition, allograft osteochondral transplantation lacks donor sources and increases the potential risk of transmitting diseases.^[^ ^5^ ^]^ Autologous osteochondral transplantations also cause secondary injuries and are only appropriate for minor cartilage defects.^[^ ^6^ ^]^ Cell‐based techniques, such as autologous chondrocyte implantation (ACI), match irregular cartilage defects, but they require two surgeries, and chondrocytes may dedifferentiate during expansion in vitro.^[^ ^7^ ^]^

In addition to the shortcomings of clinical treatments, the complex structure of osteochondral tissue and the poor proliferative capacities of chondrocytes are the fundamental reasons for the difficulty of repairing defects. The components of cartilage include water, extracellular matrices (ECMs), and chondrocytes, while the components of subchondral bone include water, mineral substances, and osteocytes.^[^ ^8^ ^]^ Osteochondral tissue is an anisotropic structure that exhibits variable properties in different directions and depths. The osteochondral characteristics, including the morphologies and phenotypes of chondrocytes, the composition of ECMs, Col orientation, and cytokines, vary with the transition from superficial cartilage to subchondral bone.^[^ ^9^ ^]^ Cartilage is divided into the superficial, intermediate, and deep regions. Chondrocytes change from horizontally arranged thin ellipsoids to vertically aligned spheres. The glycosaminoglycan (GAG) content is low in the superficial part, increases with the depth of intermediate area, and then decreases near the tidemark. Col is more abundant in the superficial and intermediate regions, while its abundance decreases near the tidemark in deep places. Moreover, Col is horizontally arranged in the superficial region, randomly arranged in the intermediate area, and vertically arranged in the deep region and calcified cartilage layer.^[^ ^10^, ^11^ ^]^ The force experienced during joint movement contributes to the Col arrangement.^[^ ^12^ ^]^ Horizontally arranged Col disperses shear forces during articulation, vertically arranged Col resists compressive forces, and randomly arranged Col experiences compressive and shear forces and resists forces from multiple directions.

Furthermore, the calcified cartilage, which serves as the transition zone for osteochondral tissue, shares common characteristics with cartilage and subchondral bone. Type II collagen (Col II) from cartilage and Col I from subchondral bone are vertically aligned and integrated into the calcified cartilage region.^[^ ^13^ ^]^ The wavy tidemark of adjacent cartilage and irregular cement line of adjacent subchondral bone reside on either side of calcified cartilage. The semi‐permeable calcified cartilage layer allows molecules less than 500 Da to enter the cartilage layer from the subchondral bone.^[^ ^14^ ^]^ This particular barrier is essential for preserving the integrity of repaired cartilage and preventing upward subchondral bone growth. With progressive deepening, the subchondral bone becomes loose and porous from dense, and bone marrow‐derived mesenchymal stem cells (BM‐MSCs) are abundantly present in the pore structures. The bone tissue also has different collagen/nanohydroxyapatite (Col/nHA) bone matrices and osteocyte arrangements for compression and tension in different directions.^[^ ^15^ ^]^ Cartilage and subchondral bone have different physiological and mechanical properties, but their functions are closely interlinked.^[^ ^16^ ^]^ The force is transmitted down through the cartilage and distributed to the subchondral bone, and the subchondral bone provides mechanical support to the cartilage through the reaction force.^[^ ^17^ ^]^

Tissue engineering has been widely studied because it is used to mimic natural structures and has the potential to achieve simultaneous repair of osteochondral tissue. Although satisfactory results have been achieved,^[^ ^18^, ^19^ ^]^ some issues still need to be addressed. Most previously developed scaffolds did not focus on reconstructing the gradient osteochondral structure, resulting in insufficient interfacial integration or a disordered hierarchical structure between the cartilage and subchondral bone.^[^ ^20^ ^]^ Therefore, inspired by natural osteochondral morphology, the gradient scaffolds with gradually varying pores, components, and osteochondrogenesis‐inducing factors were explored, which induce stem cell differentiating to osteocyte or chondrocyte. The pore mainly affects substance transport and cell metabolism. The component gradient mimics the mechanical properties of natural osteochondral structures and produces better stress stimulation. The signal gradient generated by osteochondrogenesis‐inducing factors affects the differentiation of stem cells and the maintenance of cell phenotype and regenerates osteochondral tissue similar to the natural structure at a specific location. Accordingly, the gradient scaffold increases the interface integration between the regenerated cartilage and the subchondral bone layer and promotes the integration of regenerated and natural tissue.

**SCHEME 1:**
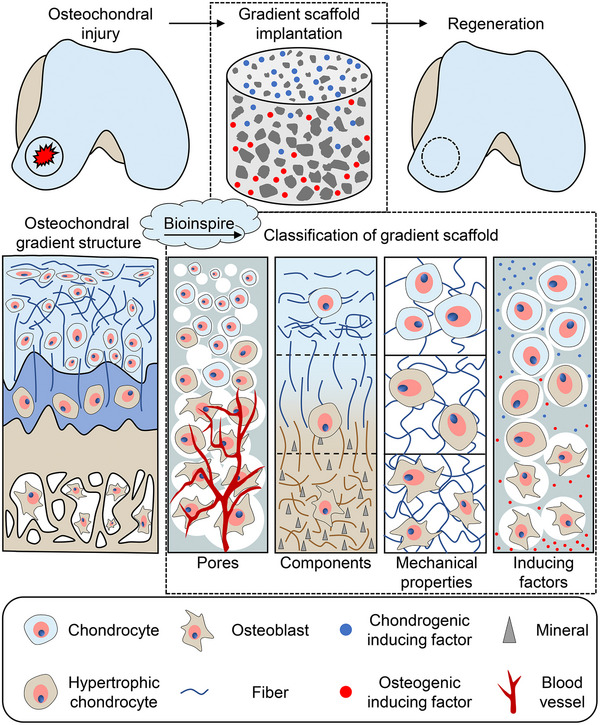
Schematic illustration of bioinspired scaffolds with gradient pores, components, mechanical properties, and inducing factors for osteochondral repair.

This review describes the gradient properties of scaffolds for osteochondral tissue engineering (Scheme 1). We emphasize the applications of bioinspired gradient scaffolds and the impact of gradient changes on repairing osteochondral defects. The current crucial issues related to osteochondral tissue engineering and future directions are discussed.

## 2 REPAIR OF OSTEOCHONDRAL DEFECTS WITH GRADIENT SCAFFOLDS

An ideal scaffold should have an appropriate degradation rate, good biocompatibility, permeability for the diffusion and exchange of nutrients and metabolites, and mechanical properties comparable to one of the normal tissues.^[^ ^21^ ^]^ Concerning the optimal degradation rate, the rate of tissue regeneration by the scaffold should be closely related to the degradation rate of materials to allow synchronous substitutions.^[^ ^22^ ^]^ The cartilage thickness and position are determined cooperatively by the positively regulated continuous chondrogenesis toward the outer surface and the negatively regulated tidemark mineralization front. However, since subchondral bone regeneration is based on the hypertrophic calcification of chondrocytes (endochondral osteogenesis),^[^ ^23^ ^]^ the degradation rate of the cartilage layer should be faster than that of the subchondral bone layer in osteochondral tissue engineering scaffolds. Biocompatibility requires that the materials and degradation products should be nontoxic and elicit an appropriate response rather than an excessive inflammatory response. Graft failure is a vital hurdle for eventual clinical applications due to low biocompatibility and an excessive immune response.^[^ ^24^ ^]^ Many PLGA degradation products lead to local over‐acidity, which is detrimental to cartilage growth. In contrast, adding magnesium hydride nanoparticles prevents the pH drop, reduces the production of cell inflammatory factors, and promotes cartilage regeneration.^[^ ^25^ ^]^

With increasing depth, the compressive and elastic moduli of cartilage increased from 0.27−1.16 to 0.71−7.75 MPa and from 0.020 ± 0.003 to 6.44 ± 1.02 MPa, respectively,^[^ ^11^ ^]^ while the mechanical properties of bones vary significantly with age and bone parts. Compact bones are more robust and stiffer longitudinally than horizontally, and the porosity and arrangement of bone trabeculae also determine their mechanical properties.^[^ ^26^ ^]^ The modulus of calcified cartilage is intermediate between articular cartilage and subchondral bone, and they form a hierarchy of mechanical properties from top to bottom that transmits forces while reducing stress concentrations at the osteochondral interface.^[^ ^27^ ^]^ Excellent scaffolds should accomplish the stress transmission between cartilage, subchondral bone, and surrounding natural tissue, thereby stimulating regeneration.^[^ ^28^ ^]^ Changes in the mechanical and architectural properties of scaffolds affect tissue growth and development by influencing biological signals, such as cell differentiation, expansion, and proliferation.^[^ ^29^ ^]^

**FIGURE 1:**
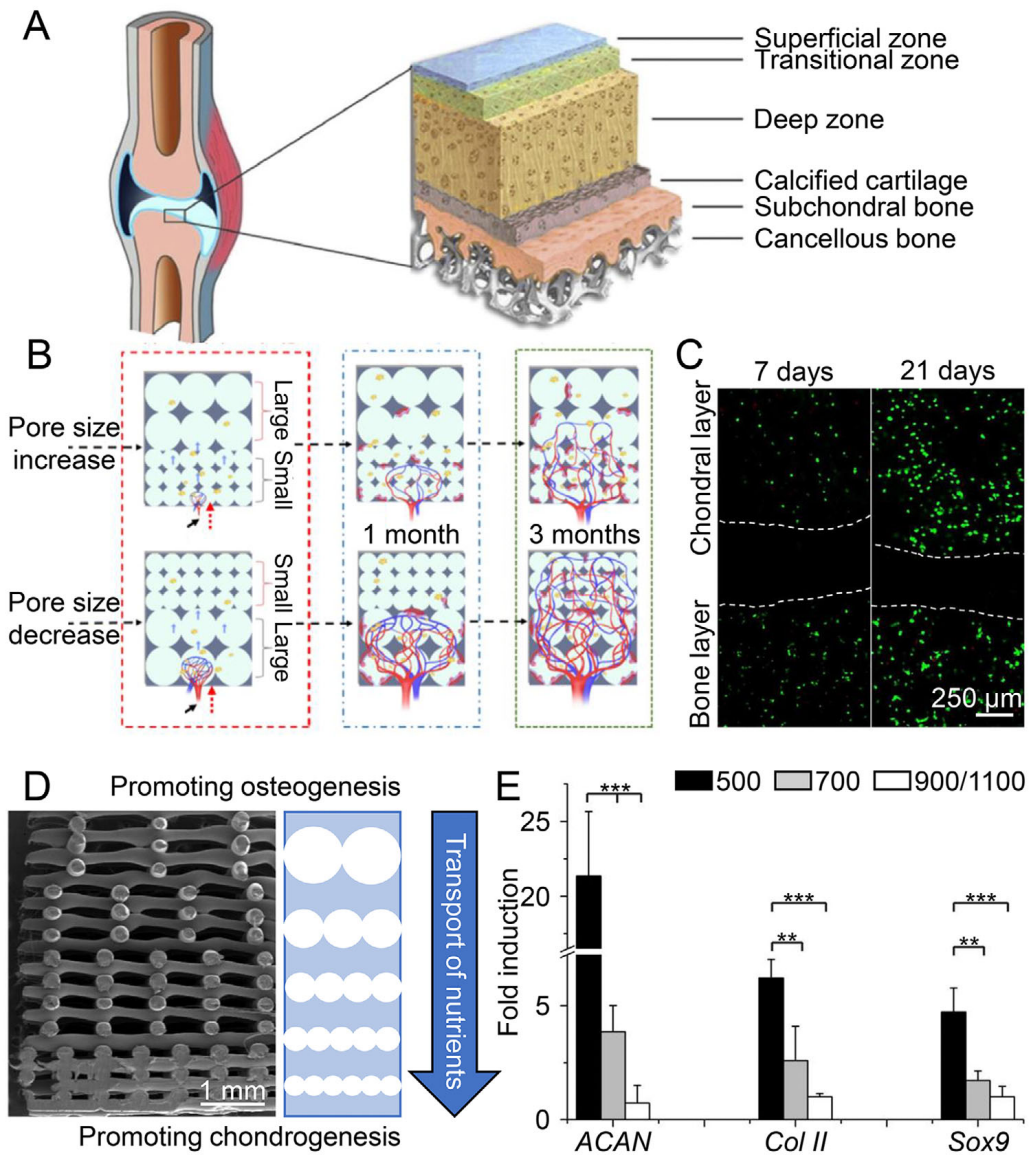
Effect of pore size on osteochondral repair. A) The natural osteochondral structure can be divided into several different tissues and simulated by scaffolds with different pore sizes. Reproduced with permission.^[^ ^23^ ^]^ Copyright 2017, Elsevier. B) Vascular infiltration in scaffolds with increasing and decreasing pore sizes. Vessels that initially grow in macropores create a more complex network, while scaffolds with small pore sizes impede vascular invasion. Reproduced with permission.^[^ ^32^ ^]^ Copyright 2020, Elsevier. C) Confocal laser scanning microscopy (CLSM) images of scaffolds seeded with cells in top and bottom layers. The cell‐free zone between the white dashed lines shows that no cells are able to pass through the calcified cartilage layer. Reproduced with permission.^[^ ^42^ ^]^ Copyright 2018, Multidisciplinary Digital Publishing Institute. D) Scanning electron microscopy (SEM) image of gradient scaffold cross‐section. The pore sizes of scaffolds affect nutrient transport. E) Differentiation of hMSCs in different pore size layers of gradient scaffolds at a genetic level. Data are represented as mean ± standard deviation (SD; **P < 0.01, ***P < 0.001). Reproduced with permission.^[^ ^46^ ^]^ Copyright 2016, John Wiley & Sons.

In brief, modulating the different physiological properties of cartilage and subchondral bone through homogeneous scaffold engineering is challenging. Nevertheless, scaffolds with gradient characteristics enable cartilage and subchondral bone layers with different physicochemical properties and mimic the native osteochondral tissue structure (Figure 1A). We summarized the pore gradient in Table 1 and the material gradient in Table 2.

**TABLE 1:** Pore gradients involved in osteochondral scaffolds.

		Characteristics of scaffold			
Type of gradient	Functions	Materials	Synthetic technology	Gradient change (from cartilage to subchondral bone)	Reference
Size	Small pore promoted chondrogenesis; Large pore promoted vascularization and osteogenesis; The interface layer separated the microenvironments of cartilage and subchondral bone regions	PEOT/PBT	3D printing	Discrete gradient, four layers: 237.7, 389.3, 509, and 538.3 μm	^[^ ^46^ ^]^
		Gelatin, fibrinogen, hyaluronic acid, anPCL	3D printing	Discrete gradient, four layers: 150, 350, 550, and 750 μm	^[^ ^34^ ^]^
		Chitosan, silk fibroin, anHA	Electrospinning	Discrete gradient, three layers: 152.6 μm, nanopore interface, and 257.3 μm	^[^ ^42^ ^]^
		nHA PCL	3D printing	Discrete gradient, three layers: 200, 500, and 900 μm	^[^ ^48^ ^]^
Shape	Square pore promoted chondrogenesis; Rhomboidal pore promoted osteogenesis	PCL	3D printing	Discrete gradient, six layers: from square to rhombus, 90°, 75°, 60°, 45°, 30°, and 15°	^[^ ^53^ ^]^
		nHA, PCL	3D printing	Discrete gradient, four layers: from square to rhombus, 90°, 45°, 30°, and 15°	^[^ ^54^ ^]^
Orientation	Surface lubrication layer promoted chondrogenesis; Vertical channel promoted cell migration and nutrient diffusion; Interface layer separated the microenvironments of cartilage and subchondral bone regions; Interconnecting pore promoted bone ingrowth	PEGDA	Freeze drying	Discrete gradient, three layers: nonporous lubricated surface, vertical channel, and interconnecting pores	^[^ ^78^ ^]^
		ACECMs, PLGA	TIPS, 3D printing	Discrete gradient, three layers: vertical channel, nonporous dense interface, and interconnecting pores	^[^ ^57^ ^]^

Abbreviations: 3D, three‐dimensional; ACECMs, acellular extracellular matrices; nHA, nanohydroxyapatite; PCL, poly(ε‐caprolactone); PEGDA, poly(ethylene glycol)‐diacrylate; PEOT/PBT, poly(ethylene oxide terephthalate)/poly(butylene terephthalate); PLGA, poly(lactic‐*co*‐glycolic acid); TIPS, thermal‐induced phase separation.

**TABLE 2:** Component gradients involved in osteochondral scaffolds.

		Characteristics of scaffold			
Type of gradient	Functions	Materials	Synthetic technology	Gradient change (from cartilage to subchondral bone)	Reference
Component	Materials suitable for cartilage or subchondral bone properties influence the migration and differentiation of MSCs; Appropriate component gradient induce the formation of calcified cartilage and promote osteochondral integration	nHA, PCL	Selective laser sintering	Discrete gradient, seven layers: nHA content from 0 to 30% in increments of 5% per layer	^[^ ^23^ ^]^
			3D printing	Discrete gradient, three layers: nHA content was 0%, 15%, 30%	^[^ ^48^ ^]^
		Chitosan, silk fibroin, nHA	Electrospinning	Discrete gradient, three layers: Chitosan existed in three layers; Silk fibroin existed in cartilage and calcified cartilage layers, gradually decreasing; nHA existed in calcified cartilage and subchondral bone layers, gradually increasing	^[^ ^42^ ^]^
		Hyaluronic acid, Col II, Col I, nHA	Iterative layering	Discrete gradient, three layers: Col II existed in cartilage layer; Hyaluronic acid existed in cartilage and calcified cartilage layers, gradually decreasing; nHA existed in calcified cartilage and subchondral bone layers, gradually increasing; Col I existed in three layers, gradually increasing	^[^ ^58^,^59^ ^]^
		Col I, nHA	Freeze drying	Discrete gradient, three layers: Col I content was 100%, 60%, 40%; nHA content was 0%, 40%, 60%	^[^ ^60^ ^]^
		Col II, CS, Col I, HA	Liquid‐phase cosynthesis	Discrete layer and continuous interface, three layers; Col II existed in cartilage and calcified cartilage layers, gradually decreasing; CS existed in the cartilage layer; Col I existed in thesubchondral bone layer; HA existed in calcified cartilage and subchondral bone layers, gradually increasing;	^[^ ^63^ ^]^
		Gelatin, β‐GP, chitosan	Freeze drying	Continuous gradient: Chitosan content was gradually decreasing; Gelatin and β‐GP content was gradually increasing	^[^ ^65^ ^]^
		nHA, sodium alginate, PAM	3D printing	Discrete gradient, three layers: nHA content was 0%, 40%, and 70%	^[^ ^102^ ^]^
Stiffness	Lower stiffness matrix promoted chondrogenesis, higher stiffness matrix promoted osteogenesis	Silk fibroin	Crosslinking and electric field	Discrete gradient, four layers: 23, 64, 107, and 133 kPa	^[^ ^69^ ^]^
			Crosslinking	Discrete gradient; three layers; 20.7 kPa, 49.5 kPa, 128.5 kPa	^[^ ^73^ ^]^
		Col I, nHA	Freeze drying	Discrete gradient; four layers; gradually increasing	^[^ ^70^ ^]^
		Hyaluronic acid, nHA, chitosan	Freeze drying	Discrete gradient; two layers; 6.9 kPa, 55.8 kPa	^[^ ^72^ ^]^

Abbreviations: 3D, three‐dimensional; Col, collagen; CS, chondroitin sulfate; MSCs, mesenchymal stem cells; nHA, nanohydroxyapatite; PAM, polyacrylamide; PCL, poly(ε‐caprolactone); β‐GP, β‐glycerophosphate.

### 2.1 Osteochondral scaffolds with pore gradients

As essential parameters for tissue engineering scaffolds, pore size and porosity are associated with angiogenesis and nutrient transport.^[^ ^30^, ^31^ ^]^ By changing the pore size and porosity of scaffold, the growth of microvessels is controlled, and the degree of vascularization is better at macropores than at tiny pores. From the growth and development perspective, the interconnected macropores provide space for the infiltration of microvessels and increase vascular branching (Figure 1B),^[^ ^32^ ^]^ improving nutrient and oxygen exchange efficiency. From the cell perspective, the larger pore size promotes the polarization of M1‐type macrophages into M2,^[^ ^33^ ^]^ which later secrete more cytokines that favor vascular growth. A gradient‐structured scaffold was developed with pore sizes of 150, 350, 550, and 750 μm from the superficial cartilage layer to the subchondral bone layer.^[^ ^34^ ^]^ The angiogenic marker CD31 was only expressed in the 750 μm layer of gradient‐structured scaffold group, which verified this gradient scaffold confined microvessels to the subchondral bone layer. Hence, gradient scaffolds construct the repaired tissue with better layer specificity than homogeneous scaffolds.

Considering the distribution of microvessels in the osteochondral tissue, chondrocytes obtain fewer nutrients and maintain their normal function, while more nutrients reach the bone layer through blood vessels.^[^ ^35^ ^]^ Inappropriate high porosity leads to vascular growth toward cartilage and the phenotypic transformation of chondrocytes into a hypertrophic form,^[^ ^36^ ^]^ representing the end‐stage chondrocytes widely existing in severe osteoarthritis.^[^ ^37^, ^38^ ^]^ With the number of hypertrophic chondrocytes increasing, cartilage loses its normal function and then develops osteoarthritis, as manifested by increased thickness, reduced elasticity, matrix calcification, and degradation.^[^ ^39^ ^]^ The calcified cartilage also inhibits the growth of blood vessels into cartilage,^[^ ^40^ ^]^ and its functions are separating the cartilage and subchondral bone microenvironments to prevent the cross‐migration of cells and promote cell growth in the respective regions.^[^ ^41^ ^]^ The chondrocytes and osteoblasts have been separated by a cell‐free region in the gradient triphasic scaffold with pores of 2.8−9.05 μm as the interfacial layer to mimic calcified cartilage structures (Figure 1C).^[^ ^42^ ^]^

Besides inhibiting vascular infiltration, tiny pores also facilitate cell aggregation, which is necessary to form and maintain the chondrocyte phenotype.^[^ ^43^ ^]^ Moreover, the alteration of pore size may indirectly lead to a local gradient in nutrient utilization during the culture and affect the differentiation of stem cells (Figure 1D). The effects of oxygen on the development and maintenance of osteochondral tissue are mainly mediated by the hypoxia‐inducible factor (HIF) pathway. Hypoxic culture conditions favor cartilage formation by promoting early expression of *N*‐calmodulin and late expression of aggregated proteoglycans,^[^ ^44^ ^]^ while hyperoxia promotes hypertrophic cells and osteoblasts.^[^ ^45^ ^]^ Chondrogenic genes transcription levels and GAG deposition increased within the small pore area of a multilayer scaffold with gradient pore sizes from 237.67 to 538.33 μm, partly due to the expression of the aggregated molecules (tenascin C, fibronectin, and *N*‐cadherin) after MSCs rapidly filled the pores and underwent aggregation (Figure 1E).^[^ ^46^ ^]^

**FIGURE 2:**
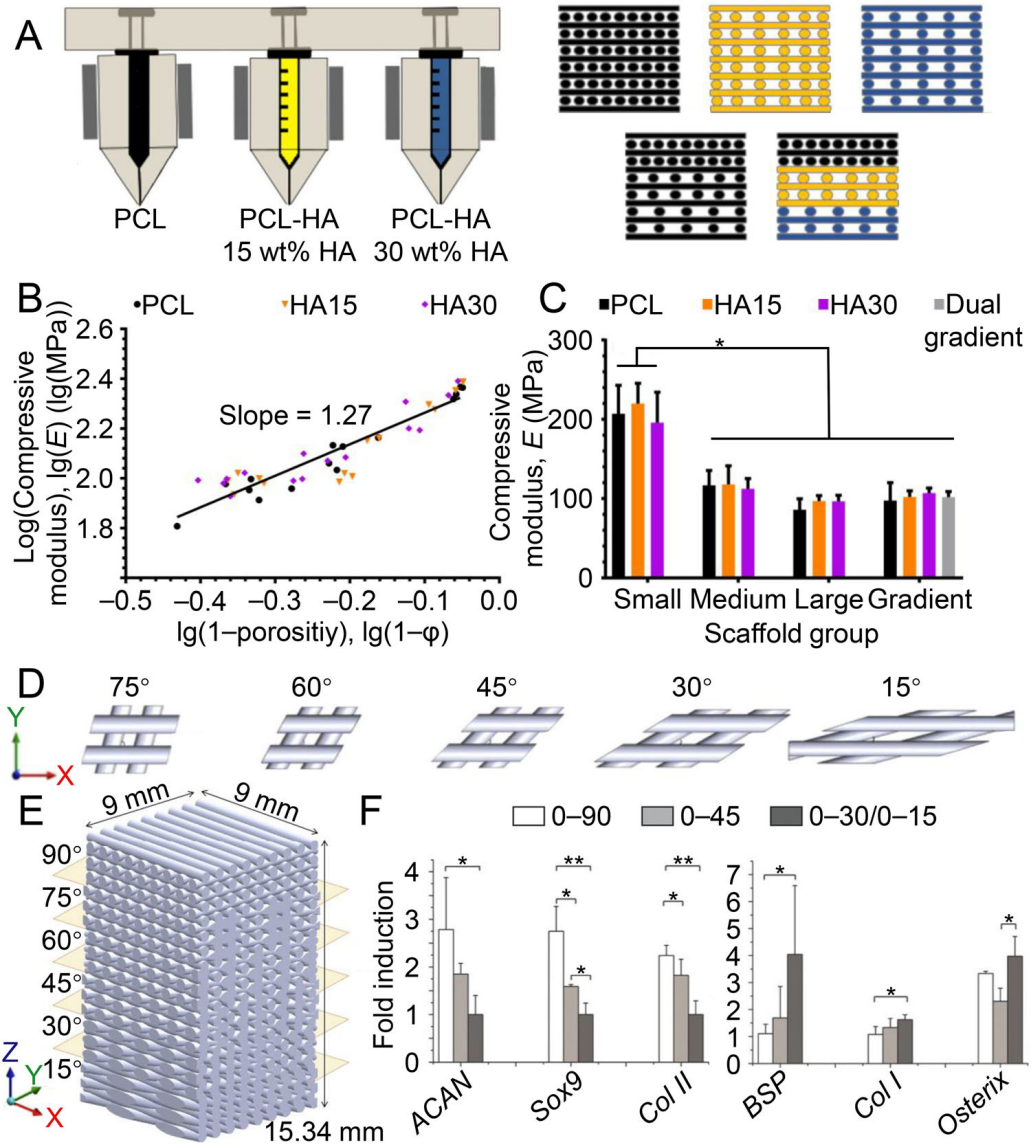
Effects of porosity and pore shape on osteochondral repair. A) Three‐dimensional (3D) printing schematic illustration for manufacturing PCL and PCL‐nHA scaffolds. B) The equation for the compressive modulus as a continuous function of precompressed porosity of the scaffolds is determined as lg(*E*) = 1.27lg(1 − *φ*) + 2.39, R2 = 0.8698. *E*, integrated modulus. C) The compressive modulus of small‐pore scaffold is significantly higher than others, while the moduli of medium‐pore, large‐pore, single‐gradient, and double‐gradient scaffolds are statistically similar. Data are represented as mean ± SD (*n* = 5; *P < 0.05). Reproduced with permission.^[^ ^48^ ^]^ Copyright 2019, Elsevier. D) Various laydown angles in *XY* plane and E) a pore‐shaped gradient scaffold consisting of six layers with different laydown angles. Reproduced with permission.^[^ ^53^ ^]^ Copyright 2020, Elsevier. F) Expression of chondrogenic and osteogenic genes in different regions of pore‐shaped gradient scaffolds. As the pore changes from square to diamond‐shaped, chondrogenic gene expression decreases while osteogenic gene expression increases. Reproduced with permission.^[^ ^54^ ^]^ Copyright 2016, John Wiley & Sons.

Pore sizes and porosities severely affect the mechanical properties of scaffolds. Pores play a significant role in the concentration of stress, especially in load‐bearing situations, where larger pores or higher porosities may disrupt the structural integrity and reduce the peak mechanical properties of materials.^[^ ^47^ ^]^ By three‐dimensional (3D) printing technology, scaffolds with gradient pore size or composition variation can be fabricated (Figure 2A), in which there was a positive correlation between compression modulus and porosity on the logarithmic scale, while the addition of HA could not significantly affect the mechanical strength of scaffolds (Figure 2B). Instead, the overall compressive strength of the gradient structure was more affected by the higher porosity fraction. Although the compressive moduli of uniform scaffolds with tiny pores were much higher than those of the uniform scaffolds with mesopores and macropores, the scaffolds with gradient pore sizes had statistically similar compression characteristics to those of uniform scaffolds with mesopores and macropores (Figure 2C).^[^ ^48^ ^]^

The overall modulus strongly depends on the region with the lowest modulus rather than the average value of modulus in each area.^[^ ^49^, ^50^ ^]^ Scaffolds with gradient pore sizes at each displacement exhibit lower compressive strength than scaffolds with uniform pore sizes.^[^ ^51^ ^]^ Both the pore and mechanical properties of scaffolds significantly affect bone growth. High porosity and macropores have sound biosynergistic effects on bone growth. The scaffolds with porosity comparable to trabecular bone porosity (70%−90%) improve cell viability and promote bone growth since interconnected pores of scaffold promote osteoconductivity and osteointegration.^[^ ^52^ ^]^ As well as macropores enhance vascularization and provide more nutrients for bone growth. On the other hand, the large pore size and high porosity may sacrifice the mechanical support of scaffold for bone growth. Hence, the porosity and mechanical strength must be balanced when designing scaffolds.

The pore shape also influences the mechanical strength and osteochondrogenesis ability of scaffold. The shape was adjusted by changing the deposition patterns between each fiber during 3D printing (Figure 2D,E). The smaller fiber laydown angle was associated with more joint arrangement asymmetry in the vertical direction, leading to rhomboid pores with lower compressive strength than square pores. However, rhomboid pores slid more easily against each other in the vertical direction, thereby increasing the deformability of scaffolds.^[^ ^53^ ^]^ Moreover, the square pores supported better chondrogenic differentiation. Meanwhile, the cells in the rhomboidal pores exhibited better osteogenic differentiation within a gradient scaffold where the fiber laydown angle transitioned from 90° to 15° (Figure 2F). Additionally, the number of HIFs increased when the pore shape changed from rhombus to square, indicating that the pore shape also affected the rate of oxygen utilization for cells.^[^ ^54^ ^]^

### 2.2 Osteochondral scaffolds with component gradients

Various natural and synthetic materials are currently used to develop osteochondral tissue engineering scaffolds with satisfactory therapeutic results. Like biological tissue structures, natural materials present outstanding biocompatibility and promote cell adhesion. Nevertheless, synthetic materials show marked superiority in the controllability of degradation rate, mechanical strength, and a wide range of sources. Given the gradient properties of natural osteochondral composition, gradient changes in the scaffold component serve an essential role in enhancing the biomimetic effects.

**FIGURE 3:**
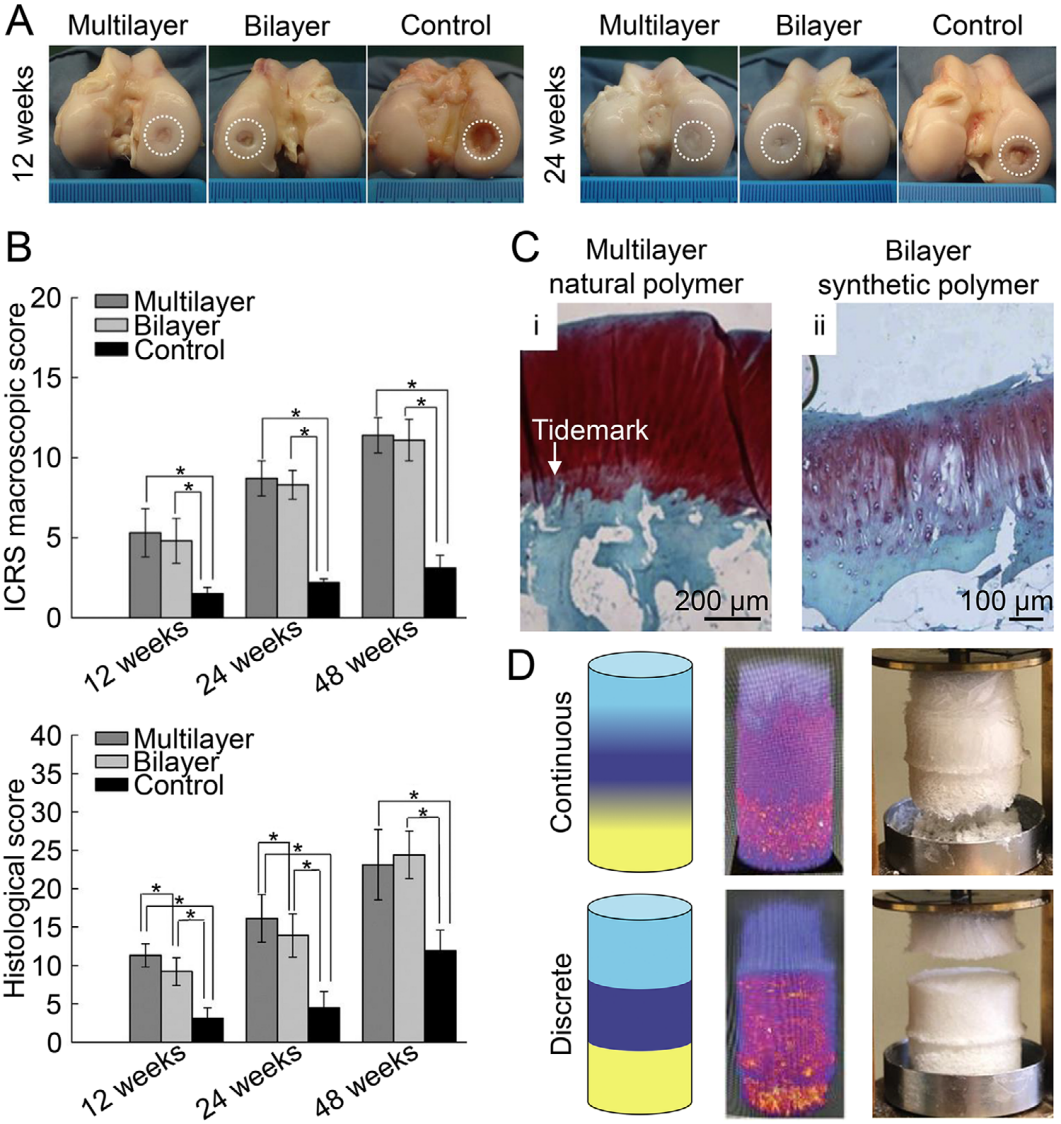
The multi‐layered scaffolds had better osteochondral repair effects. A) Macrophotographs of different groups at 12 and 24 weeks after scaffold implantation in defect. The white dashed circle represents the defect area. B) ICRS macroscopic and histological scores of cartilage repair. Data are represented as mean ± SD (*P < 0.05). ICRS, International Cartilage Repair Society. Reproduced with permission.^[^ ^57^ ^]^ Copyright 2018, American Chemical Society. C) At 48 weeks, the multilayer natural polymer scaffold group forms the tidemark and repaired the defect with hyaline cartilage tissue (i), while no tidemark formed in the bilayer synthetic polymer scaffold group, and the repaired tissue is fibrocartilage (ii). Reproduced with permission.^[^ ^59^ ^]^ Copyright 2016, Elsevier. D) Qualitative reconstruction of intensity mapping of continuous gradient and discrete gradient scaffolds after micro‐CT scanning and images of disintegrating scaffolds after delamination tests. Reproduced with permission.^[^ ^63^ ^]^ Copyright 2022, John Wiley & Sons.

Two types of gradient scaffolds are currently available: discrete and continuous. Commonly, discrete gradient scaffolds are biphasic (cartilage and subchondral bone layers), triphasic (cartilage, calcified cartilage, and subchondral bone layers), or multi‐layered, including different materials or different contents residing in the various layers. Biphasic scaffolds affect the regeneration of osteochondral defects based on the inherent ability of the different materials to promote osteogenesis or chondrogenesis, respectively. But triphasic scaffolds more closely resemble the natural structure for adding a calcified cartilage layer that overlaps from the component of cartilage to the subchondral bone, thereby improving the repair of osteochondral defects.^[^ ^55^, ^56^ ^]^ The calcified cartilage layer inhibits upward invasion of the subchondral bone while providing better cartilage support, and a multilayer scaffold with a calcified cartilage layer typically displays better surface cartilage coverage (Figure 3A), a better quality of cartilage repair (Figure 3B), subchondral bone filling, and osteochondral integration.^[^ ^57^ ^]^

Gradient scaffolds tend to achieve better biometrics by selecting more appropriate components based on the characteristics of cartilage and subchondral bone. A component gradient scaffold with Col I and nHA as a bone layer, Col I and hyaluronic acid as calcified cartilage layer, Col I, Col II, and hyaluronic acid as cartilage layer possessed outstanding performance in osteochondral repair.^[^ ^58^ ^]^ Moreover, neovascularization in the subchondral bone area and tidemark, as an essential marker for the regeneration of calcified cartilage, were observed after implanting this multilayer bionic Col‐based scaffold into caprine joints. Compared with the synthetic bilayer scaffold of poly(lactic‐*co*‐glycolic acid) (PLGA) and calcium sulfate, its tidemark provided long‐term stability to the cartilage and avoided degenerative changes in the joint due to bone overgrowth, thereby exhibiting enhanced cartilage and subchondral bone regeneration (Figure 3C).^[^ ^59^ ^]^ Notably, the acellular scaffolds with specific component gradients support a suitable microenvironment for layer‐specific tissue regeneration without an excessive inflammatory response, possessing broad clinical application prospects.

A clinical trial involved the implantation of 13 patients with gradient scaffolds in which the cartilage layer consisted of Col I, the transition layer consisted of 60% Col I and 40% nHA, and the subchondral bone layer consisted of 30% Col I and 70% nHA.^[^ ^60^ ^]^ Magnetic resonance imaging (MRI) evaluation showed good stability and no scaffold detachment at 6 months, but some patients observed subchondral bone changes and undifferentiated inhomogeneous signals. Two cases showed subchondral bone formation and complete scaffold reabsorption. However, this clinical trial did not compare the therapeutic efficacy of gradient scaffolds with others. Col recruited cells to defects, and MSCs migrated from regions with low Col concentrations to those with high Col concentrations.^[^ ^61^ ^]^ This migration behavior is conducive to cell distribution and the subsequent regeneration of osteochondral defects.

Significantly, the discrete connections between the phases may form weak mechanical interfaces, resulting in structural failure under loading conditions.^[^ ^62^ ^]^ The continuous gradient scaffold provided better articulation between layers than the discrete gradient scaffold. After separation tests, the continuous gradient scaffold collapsed at the bottom of scaffold, while the discrete gradient scaffold failed in the overlap area (Figure 3D).^[^ ^63^ ^]^ Besides, continuous gradient scaffolds also have a better biomimetic effect due to the constant gradient change. A microfluidic printing head with a hybrid unit was used to extrude two bioinks and synthesize a continuous gradient scaffold simultaneously.^[^ ^64^ ^]^ The scaffolds with a continuous gradient of chitosan, sodium β‐glycerophosphate (β‐GP), and gelatin have been verified to effectively simulate the natural physiological hierarchical structure of cartilage in physical property assessment (water absorption, porosity, and degradation rate) and cell experiments (adhesion and proliferation).^[^ ^65^ ^]^

**FIGURE 4:**
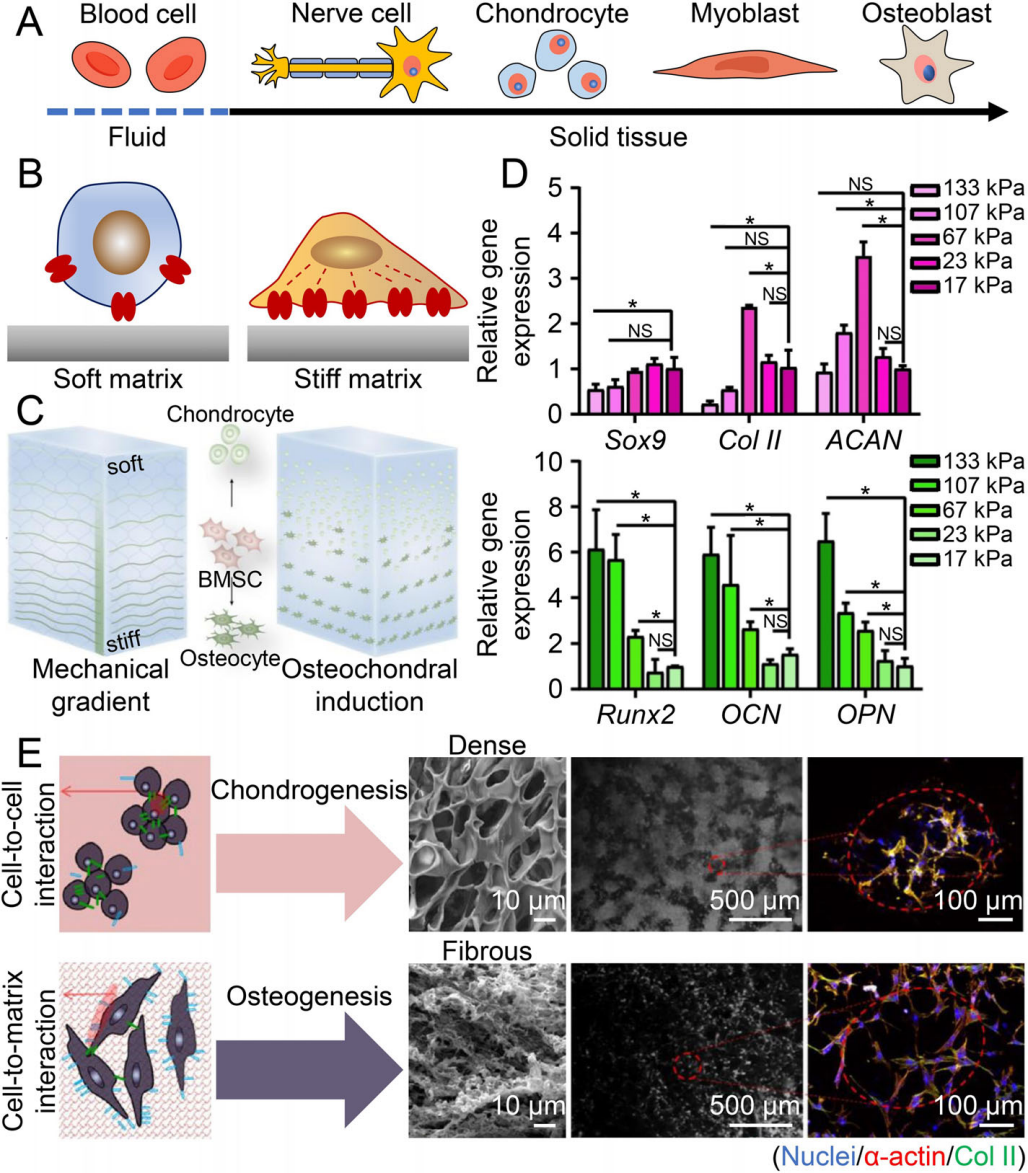
Effects of stiffness and roughness on osteochondral repair. A) Different matrix stiffnesses promote multilineage differentiation of stem cells. B) On a soft matrix, stem cells exhibit a small spreading morphology, unclear actin cytoskeleton, low level of lamin‐A, and detached focal adhesion complexes. Stem cells cultured on a stiff matrix show a small spreading morphology, significant stress fibers, high levels of lamin‐A, and strong focal adhesion complexes. C) The stiffness gradient hydrogel induces chondrogenic or osteogenic differentiation of BM‐MSCs and forms a structure similar to native osteochondral. D) Transcription levels of chondrogenic and osteogenic genes of BMMSCs in hydrogels with different stiffness. Data are represented as mean ± SD (*P < 0.05), NS means no significance. Reproduced with permission.^[^ ^69^ ^]^ Copyright 2020, Springer‐Verlag GmbH and Co. KG. E) Cell‐to‐cell interaction assay results are compared between dense‐ and nanofibrous‐surface scaffolds analyzed from the SEM and CLSM images. Reproduced with permission.^[^ ^43^ ^]^ Copyright 2019, SAGE Publications.

The viscoelasticity and stiffness of matrix are related to the differentiation of stem cells.^[^ ^66^, ^67^ ^]^ In elastic materials, the elasticity of matrix provides resistance to cell expansion, thereby inhibiting cell proliferation. In viscoelastic materials, rapid stress relaxation of the matrix permits cell expansion and thus promotes proliferation and ECM production. A matrix close to the elastic moduli of the brain, cartilage, muscle, and bone directly induces the differentiation of stem cells, especially MSCs, into nerve cells, chondrocytes, myoblasts, and osteoblasts (Figure 4A). Stem cells sense matrix stiffness and regulate the number of actin filaments and focal adhesion assemblies to produce deformation (Figure 4B). The forces of cell−matrix interaction, transmitted through the cytoskeleton to the nucleus, regulate the lamin‐A expression and ultimately influence the differentiation phenotype of stem cells. These behaviors are mainly influenced by the focal adhesion kinase (FAK), yes‐associated protein (YAP), and transforming growth factor‐β (TGF‐β) pathways.^[^ ^68^ ^]^ Xu et al. used *β*‐sheet‐rich silk nanofibers to form a gradient construct with increasing stiffness through crosslinkers and electric fields, divided into four parts, each with average stiffness values of 133, 107, 64, and 23 kPa, respectively.^[^ ^69^ ^]^ Although cell−matrix interactions may alter the stiffness of hydrogels, hydrogels with gradient stiffness still induce osteocytes and chondrocytes with a similar distribution to natural osteochondral tissue (Figure 4C). The basal hydrogel with a stiffness of 17 kPa could not induce the chondrogenic and osteogenic differentiation of BM‐MSCs, while the hydrogels with increasing stiffness showed a different induction tendency (Figure 4D). A higher stiffness matrix promotes stem cell osteogenesis, whereas a lower stiffness matrix promotes chondrogenesis.^[^ ^70^ ^]^ The surface roughness of a biomaterial matrix determines the amount of absorbed protein, and the anchoring points encountered during the cell attachment decide their final shape and degree of diffusion. Furthermore, cell shape regulates cell fate, as rounded BM‐MSCs tend to differentiate into the chondroblast lineage, while the expanded shape leads to osteogenic differentiation (Figure 4E).^[^ ^49^, ^71^ ^]^

Material selections or content changes develop osteochondral scaffolds with mechanical property gradients. Mixing chitosan with hyaluronic acid to form a soft matrix promoted the expression of *Col II*, while incorporating chitosan with nHA to create a stiff matrix promoted the expression of *OCN*.^[^ ^72^ ^]^ A protein/biosilica scaffold with gradient mechanical properties was designed by biomineralizing the composites with the concentration gradient of selective peptide‐R5 in vitro. For instance, with the rising silicification concentration, the compressive modulus increased from 20.7 ± 5.0 to 128.5 ± 16.4 kPa, along with enhanced osteogenesis capacity.^[^ ^73^ ^]^ Scaffolds with mechanical property gradients are tissue‐adaptive and facilitate force transmission between cartilage and subchondral bone or between normal and repaired tissue, thus avoiding layer misalignment of the regenerated tissue and promoting integration of the regenerated tissue with its surroundings.

### 2.3 Osteochondral scaffolds with pore and component gradients

As mentioned above, both the pores and materials of scaffolds significantly impact the repair of osteochondral injuries. Hence, considering both pore characteristics and material composition in the scaffold design may lead to a synergistic effect on osteochondral tissue engineering. In other words, different materials should be selected depending on the diverse needs of subchondral bone and cartilage, and then the pore characteristics of scaffolds should be designed according to the natural tissue structure to achieve biomimicry in terms of physical properties, structural characteristics, and others.

**FIGURE 5:**
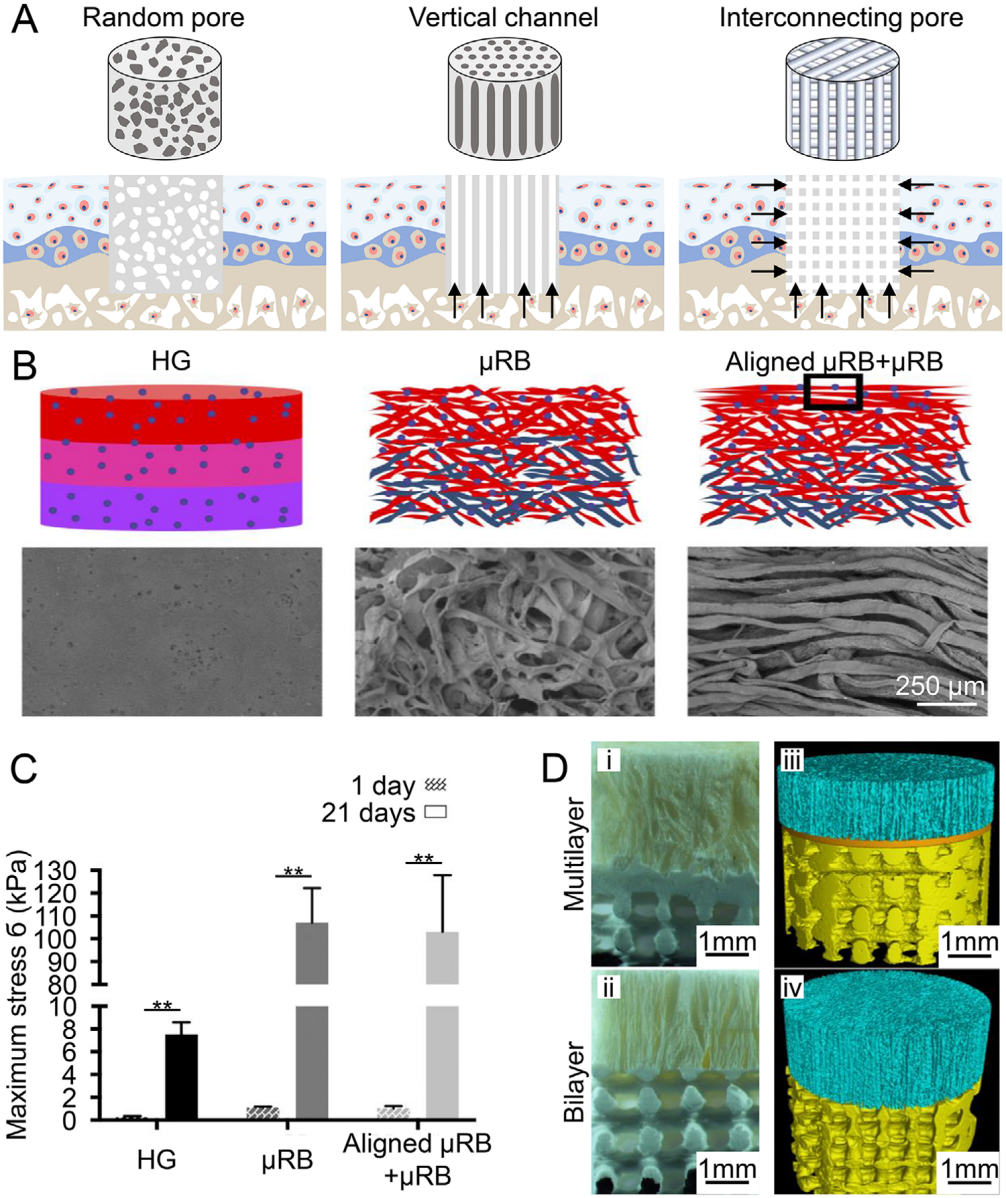
Effects of pore and material arrangement on osteochondral repair. A) Schematic diagram of scaffold structures with different pore arrangements. The arrows indicate pathways of endogenous cell migration and nutrient transport. B) SEM images of hydrogel (HG), microribbon (μRB), and aligned μRB individual layers demonstrate nanoporosity in HG layers and macroporosity in μRB layers. C) The interfacial shear strength of μRB cartilage tissues is > 10‐fold higher than that of HG tissue. Data are represented as mean ± SD (**P < 0.01). Reproduced with permission.^[^ ^77^ ^]^ Copyright 2020, Elsevier. D) Digital and micro‐CT reconstructed images of multilayer (i and iii) and bilayer scaffolds (ii and iv). The interface layer separates the microenvironments of cartilage and subchondral bone regions. The vertically aligned pore of cartilage layer intercommunicates with the large pore of subchondral bone layer, which allow nutrient and cell transport through the interface. Reproduced with permission.^[^ ^57^ ^]^ Copyright 2018, American Chemical Society.

Well‐aligned scaffolds have suitable mechanical properties and good biocompatibility.^[^ ^74^ ^]^ The oriented channels in the scaffolds contribute to cell migration and infiltration, as well as fluid flow and nutrient diffusion in vivo (Figure 5A). During the tissue remodeling process, the cartilage regeneration of radially aligned scaffolds was dramatically faster than that of randomly and axially aligned scaffolds.^[^ ^75^, ^76^ ^]^ As above, the addition of aligned fiber to the top of a random fiber layer improved the mechanical property of scaffold (Figure 5B,C) and restored the superficial region phenotype of articular cartilage, as proven by high Col production and superficial region proteins.^[^ ^77^ ^]^ Besides, the alignment of infiltrating cells was dictated by the pore architecture of the layer, as proven by the random arrangement of cells in the dense superficial cartilage layer and subchondral bone layer with interconnected pores, while the vertical arrangement of cells in the medial cartilage layer with columnar pores in a trilayer scaffold.^[^ ^78^ ^]^ Chen et al. used electrospinning to fabricate scaffolds with superficial parallel, intermediate crossed, and deep vertical fibers, where the cultured MSCs were topographically influenced to produce anisotropic tissue fibers.^[^ ^79^ ^]^

Considering the hierarchical specificities of both pore and composition structure in the osteochondral tissue, Jia et al. synthesized a multilayer scaffold in which acellular extracellular matrices (ACECMs) were utilized as the cartilage layer, and PLGA/β‐tricalcium phosphate (β‐TCP; β‐Ca_3_(PO_4_)_2_) was applied as the middle and bone layers.^[^ ^57^ ^]^ From top to bottom, there was a vertical microtubule structure with a diameter of 84.2 ± 20.7 μm, a dense middle layer, and a bone layer with a diameter of 450.5 ± 47.2 μm. (Figure 5D). The multi‐layered scaffold showed better repair effects at each time point than the scaffold without an intermediate layer. The vertically oriented microtubule structure was conducive to the recruitment, adhesion, and nutrient metabolism of MSCs, and the dense middle layer was designed to resemble the tidemark to support the formation of columnar‐like cell clusters.

The nanoscale matrix or the increased roughness of material enhances cell adhesion, thereby prolonging the cell morphology and promoting osteogenic differentiation.^[^ ^43^ ^]^ When MSCs were seeded in the alginate/hyaluronic acid hydrogel and combined with a Col membrane, alcian blue (AB)‐positive staining in the hydrogel layer showed that MSCs differentiated into cartilage and secreted a large amount of GAG. Moreover, extensive alizarin red‐positive staining was observed on the surface of Col membrane, indicating that MSCs migrated, adhered to Col membrane, and differentiated into osteoblasts.^[^ ^80^ ^]^

### 2.4 Osteochondral scaffolds with osteochondrogenesis‐inducing factor gradients

As one of the three elements of tissue engineering, inducing factors play a vital role in osteochondrogenesis because they regulate cell proliferation, migration, and differentiation by affecting various signaling pathways.^[^ ^81^, ^82^ ^]^ Studies have shown that the intra‐articular injection of kartogenin, a small‐molecule drug with chondrogenesis capability, failed to exert a long‐term effect on cartilage repair as it rapidly entered the circulation or was degraded.^[^ ^83^ ^]^ Consequently, many factor release strategies have been extensively developed to enhance the long‐term impact and induce cartilage and subchondral bone formation, involving a single factor, multiple factors, and the hierarchical control of factor release.^[^ ^84^ ^]^ Many studies have investigated the incorporation of various induction factors in different scaffold layers, thereby inducing the regeneration of osteochondral defects. However, the biomimetic effect could be significantly considered by developing an inducing factor gradient corresponding to the anisotropic characteristics of osteochondral tissue. Table 3 lists the inducing factors with discrete or continuous gradients used in scaffolds and describes their roles in articular cartilage regeneration and maintenance.

**TABLE 3:** Induction factors loaded into multilayer gradient scaffolds.

Induction factor	Discrete or continuous gradient	Induction effect	Performance	Reference
TGF‐β	Continuous gradient	Chondrogenic differentiation	Promoting integration with surrounding cartilage Promoting GAG deposition and Col II secretion Promoting cell adhesion Upregulating expression of *Sox9*, *ACAN*, and *Col II*	^[^ ^90^, ^91^, ^93^, ^94^, ^105^ ^]^
BMP	Continuous gradient	Osteogenic differentiation	Promoting subchondral bone growth Increasing ALP activity Promoting mineralization Promoting osteocalcin secretion Promoting cell adhesion Upregulating expression of *Runx2*, *OCN*, *ALP*, and *Col X*	^[^ ^90^, ^91^, ^93^, ^94^, ^96^, ^105^ ^]^
β‐TCP	Continuous gradient	Osteogenic differentiation	Promoting mineralization Upregulating expression of *Runx2* and *OPN*	^[^ ^104^ ^]^
nHA	Discrete gradient, continuous gradient	Osteogenic differentiation	Promoting mineralization Promoting subchondral bone growth Promoting tidemark formation Promoting osteoblast growth Promoting integration with surrounding bone Upregulating expression of *OCN*, and *Col I*	^[^ ^23^, ^91^, ^102^, ^107^ ^]^
Bioglass	Continuous gradient	Osteogenic differentiation	Promoting mineralization Promoting secretion of Col I and OCN Increasing ALP activity Upregulating expression of *Runx2*, *ALP*, *OCN*, and *Col I*	^[^ ^105^, ^109^ ^]^
CS	Continuous gradient	Chondrogenic differentiation	Promoting Col II secretion Upregulatinge expression of *ACAN*, and *Col II* Promoting chondrocyte growth	^[^ ^104^, ^105^, ^107^ ^]^
SDFs	Continuous gradient	Concentration dependence	Promoting ALP and OPN secretion Upregulating expression of *ALP*, *Runx2*, *OCN*, and *OPN*	^[^ ^110^ ^]^
Insulin	Discrete gradient	Concentration dependence	Promoting GAG deposition and Col II secretion	^[^ ^113^ ^]^
β‐GP	Discrete gradient	Osteogenic differentiation	Promoting mineralization	^[^ ^113^ ^]^
TUDCA	Discrete gradient	Concentration dependence	Low concentration: Upregulating expression of *Col I*, *Runx2*, *ALP*, and *OCN* Promoting secretion of Col II and OCN High concentration: Upregulating expression of *Col II* and *ACAN* Promoting Col II secretion	^[^ ^115^ ^]^

Abbreviations: ALP, alkaline phosphatase; BMP, bone morphogenetic protein; Col, collagen; CS, chondroitin sulfate; GAG, glycosaminoglycan; nHA, nanohydroxyapatite; SDFs, simulated body fluids; TGF‐β, transforming growth factor‐β; TUDCA, taurodeoxycholic acid; β‐GP, β‐glycerophosphate; β‐TCP, β‐tricalcium phosphate.

#### 2.4.1 Osteochondral scaffolds with gradient growth factors

The TGF‐β superfamily includes TGF‐β, bone morphogenetic protein (BMP), activin/inhibin, and growth differentiation factors (GDFs), which regulate various cell functions and significantly influence articular cartilage homeostasis and repair.^[^ ^81^ ^]^ Among them, TGF‐β and BMP are the most widely used growth factors in osteochondral tissue engineering. TGF‐β signaling is transmitted through type II receptor, which recruits and phosphorylates type I receptor, activating Smad2 and Smad3. Phosphorylated Smad2/3 interacts with Smad4 to translocate to the nucleus and initiate target gene transcription.^[^ ^85^ ^]^ TGF‐β has a stimulatory effect at the early stage of chondrogenesis, promoting cartilage matrix synthesis, cell proliferation, and upregulation of chondrogenic‐specific genes, such as *Sox9*, aggregative proteoglycan, and *Col II*. Moreover, it inhibits the terminal differentiation of hypertrophic chondrocytes by suppressing the expression of matrix metalloproteinase‐13, *Col X*, osteocalcin, and vascular endothelial growth factor (*VEGF*).^[^ ^86^, ^87^ ^]^

BMPs are also involved in diverse phases of skeletal development, but unlike Smad2/3 in the TGF‐β pathway, Smad1/5/8 is responsible for BMP signaling.^[^ ^88^ ^]^ The relationship between BMP signaling and the expression of SOX family members is a crucial regulator of chondrogenesis,^[^ ^87^ ^]^ and BMPs induce the differentiation of stem cells into osteoblasts or enhance overall osteogenic activity. This phenomenon accelerates bone repair while promoting cell communication between osteoblasts and endothelial cells to initiate angiogenesis, leading to osteogenic differentiation.^[^ ^89^ ^]^

TGF‐β1 and BMP‐2 were encapsulated into PLGA microspheres and poured into molds at different rates to form scaffolds with reverse gradients of TGF‐β1 and BMP‐2.^[^ ^90^ ^]^ The gradient scaffolds had more uniform tissue regeneration and regenerated thicker cartilage layers than scaffolds without growth factors. However, no significant differences were observed in new bone formation or mineral deposition between the groups, and only the trabeculae in the gradient group was generally thicker than that of scaffold without growth factor. Accordingly, adding BMP‐2 may exhibit a more negligible effect on bone growth than mechanical loading. NHA was also added to the system to promote subchondral regeneration. After implantation in vivo, the gradient scaffold groups demonstrated good repair of osteochondral defects, such as cartilage surface filling, subchondral bone inward growth, good mineralization, and integration of the repair boundary with the surrounding tissue. Furthermore, abundant GAG deposition was observed in the cartilage layer of TGF‐β1 and BMP‐2 groups. Nevertheless, the repair of subchondral bone layer in the BMP‐nHA group was not significantly better than that in the nHA group.^[^ ^91^ ^]^

Unexpectedly, the BMP and nHA composites produced poor synergism. The total BMP release in the PLGA/nHA microsphere group was significantly lower than that in the PLGA microsphere group, and the osteogenic differentiation activity was lower in the former. This may have been due to mineral incorporation disrupting the polymer network, resulting in poor protein retention during fabrication.^[^ ^92^ ^]^ Alternatively, the nanopore of nHA is detrimental to protein release. The high binding affinity of protein for nHA may further limit protein release, which occurs only if the surface area of microspheres expands due to fracture, leading to another rupture or release.^[^ ^82^ ^]^

Strategies for introducing inducing factor gradients are diverse. A tapered gradient chitosan‐gelatin hydrogel/PLGA scaffold delivered both TGF‐β1 and BMP‐2 microspheres.^[^ ^93^ ^]^ Compared with TGF‐β1 and BMP‐2 unloaded or single‐gradient scaffolds, the dual‐gradient scaffolds induced BM‐MSCs differentiation and demonstrated the excellent repairability of osteochondral defects. When TGF‐β3 and BMP‐2 were covalently bound to poly(ε‐caprolactone) (PCL) scaffolds, the ability to promote differentiation in vitro was superior to that of the soluble form in the cell culture medium. Nevertheless, the chondrogenic and osteogenic differentiation competence of scaffolds with TGF‐β3 and BMP‐2 reverse gradients were not superior to bare PCL scaffolds, with differences observed only in the top and bottom layers of gradient scaffolds.^[^ ^94^ ^]^ The lack of difference may be due to the short culture time in vitro or the low total amount of TGF‐β3 and BMP‐2 loaded on the gradient scaffold. Considering the ability of growth factors to bind heparin, photo‐crosslinked heparin‐alginate hydrogels were developed with a reverse gradient of TGF‐β1 and BMP‐2.^[^ ^95^ ^]^ The BM‐MSCs underwent osteogenic and chondrogenic differentiation during two weeks of culture. The expression of alkaline phosphatase (ALP) and GAG deposition was significantly increased as the BMP‐2 and TGF‐β1 concentrations increased, respectively.

**FIGURE 6:**
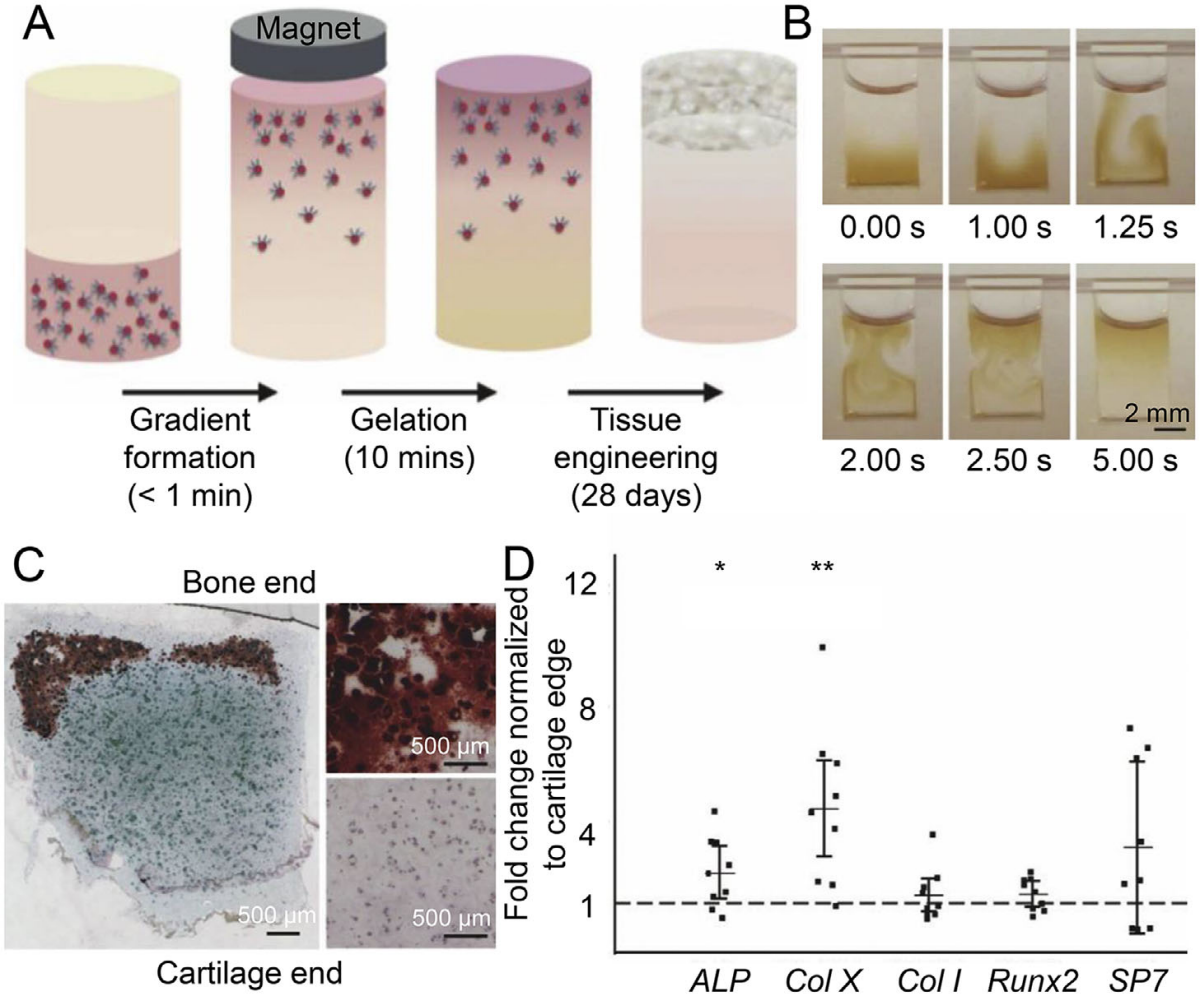
Effect of gradient BMP2 concentrations on osteochondral repair. A) Glycosylated superparamagnetic iron oxide nanoparticle (SPION) forms gradients in a magnetic field, and when cocultured with MSCs in a hydrogel, it induces engineered osteochondral structures. B) SPION forms a smooth continuous gradient in the presence of a magnetic field. C) Alizarin Red S (ARS) staining and AB staining shows calcium deposits at the bone end of engineered tissue. D) Expression of osteogenic genes in bone area compared to cartilage area. Data are represented as mean ± SD (*n* = 3; *P < 0.05, **P < 0.01). Reproduced with permission.^[^ ^96^ ^]^ Copyright 2018, Elsevier.

A unique BMP‐2 release strategy was developed based on glycosylated superparamagnetic iron oxide nanoparticles to load BMP‐2 onto agarose hydrogels, followed by forming a BMP‐2 gradient by the external application of a magnetic field (Figure 6A,B).^[^ ^96^ ^]^ BMP‐2 was continuously released for 28 days, and a cartilage area rich in Col II and GAG was observed in the produced tissue after culturing in vitro. Col X was mainly located at the interface between bone and cartilage, and minerals were widely distributed in the bone area (Figure 6C). Moreover, osteogenic genes were upregulated at the subchondral bone end compared to the cartilaginous end (Figure 6D). A magnetic susceptibility‐like BMP‐2 gradient induced a sharp transition separating the subchondral bone from the hyaline cartilage. This may cause by the need to reach a BMP‐2 threshold to initiate and maintain tissue mineralization, creating a positive feedback loop in which the deposited minerals further stimulate osteogenesis.

#### 2.4.2 Osteochondral scaffolds with gradient minerals

Bioactive glasses, mainly composed of oxides of Si, Na, Ca, and P, have been verified to stimulate bone regeneration, mainly due to their dissolution products, including soluble silica and calcium ions, which stimulate osteoblasts to produce bone matrix.^[^ ^97^ ^]^ Bioglass microfibers strengthen the mechanical strength and adhesion of scaffolds and the anchorage between scaffolds and osteochondral tissue.^[^ ^98^ ^]^ Owing to high osteoconductivity and osteoinductivity, β‐TCP is widely used as a bone graft substitute. Local acidification of osteoclasts may dissolve β‐TCP and cause it to be absorbed by the cells and replaced by new bone.^[^ ^99^ ^]^ Gradient amorphous calcium phosphate in the PLGA disk enables the spatial tissue commitment of adipose‐derived mesenchymal stem cells (A‐MSCs) by increasing the expression of *Sox9* in the cartilage layer and *OCN* in the bone layer.^[^ ^100^ ^]^ HA (Ca_10_(PO_4_)_6_(OH)_2_) is present in 70% of human bones, whereas water is present in 5%, and organic materials are present in 25%. NHA has good biocompatibility, appropriate biodegradability, and high osteoconductivity and induces BM‐MSCs to differentiate into osteoblasts, thereby accelerating subchondral bone and osteochondral interface formation. In addition, nHA combines with other biomaterials to improve bone mineralization.^[^ ^101^, ^102^, ^103^ ^]^

Since minerals mainly induce osteogenic differentiation of MSCs, some substances that promote chondrogenesis were added to the system to create a double gradient of chondrogenesis and osteogenesis, effectively repairing osteochondral defects. Chondroitin sulfate (CS) and β‐TCP were encapsulated into PLGA microspheres and poured into the mold at different speeds to form a uniform scaffold of CS or β‐TCP and scaffolds with a reverse gradient of CS and β‐TCP.^[^ ^104^ ^]^ According to the in vitro culture results, the CS group showed higher GAG deposition and hydroxyproline secretion, and the β‐TCP group showed higher mechanical strength. In addition, CS and bioglass encapsulated in scaffolds accelerated the cell secretion of ECM components.^[^ ^105^ ^]^ The gradient group showed higher transcription levels of some osteogenic and chondrogenic markers at early time points, which may depend on the faster maturation of BM‐MSCs into cartilage‐like and osteoblast‐like cells in the gradient group. The main repair effects observed for CS and minerals were most likely due to the enhanced initial interaction between the compositions and cells. When MSCs grew faster into functional cartilage‐like and osteoblast‐like cells, they secreted the required ECMs earlier, thus improving the repair efficacy and quality of osteochondral defects.

**FIGURE 7:**
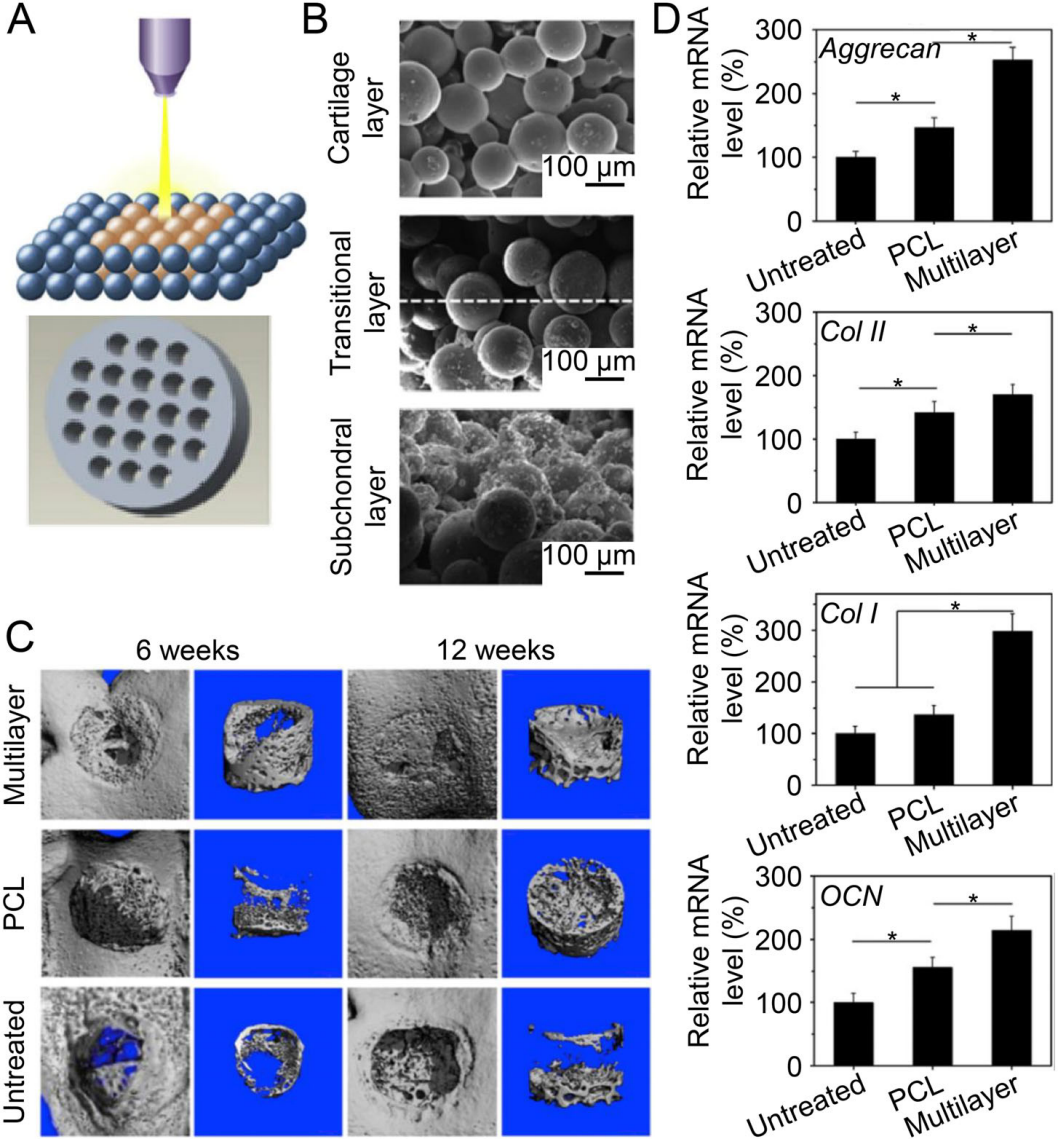
Effect of gradient nHA content on osteochondral repair. A) Schematic diagram of fabricating multilayer scaffolds by selective laser sintering technique. B) SEM images of different layers of nHA gradient scaffold. C) Micro‐CT reconstruction of regenerated subchondral bone at 6 and 12 weeks after scaffold implantation. D) Analysis of genes related to chondrogenesis and osteogenesis in regenerated tissue at 12 weeks post‐surgery. Data are represented as mean ± SD (*n* = 3; *P < 0.05). Reproduced with permission.^[^ ^23^ ^]^ Copyright 2017, Elsevier.

Graded nHA concentrations and barriers at the bone−cartilage interface are more likely to reconstruct osteochondral defects effectively.^[^ ^106^ ^]^ The gradient nHA−CS hydrogel regenerated the tidemark, hindered the microvasculature in the cartilage layer, regenerated subchondral bone with a higher bone mineral density, increased the ratio of bone volume to total defect volume and the trabecular thickness, and enhanced the integration with the surrounding normal tissues.^[^ ^107^ ^]^ During repair process, nHA is degraded and spreads to the surrounding microenvironments, where it is taken up by stem cells and induces differentiation of stem cells to complete the reconstruction.^[^ ^108^ ^]^ Du et al. sintered PCL microspheres with gradient contents of nHA together to form an osteochondral scaffold (Figure 7A,B). The gradient nHA accelerated the early regeneration of subchondral bone, enhanced the stable fusion of new bone and natural bone, and provided full anchorage to the graft in the osteochondral defects (Figure 7C). Moreover, they observed abundant cartilage tissue production and gradual tidemark formation at 6 weeks. At 12 weeks, the tidemark became clear and pushed outward, thinning the cartilage at a normal thickness and transferring it to the defect surface. In addition, the gradient scaffold group transcribed chondrogenic and osteogenic genes at higher levels (Figure 7D).^[^ ^23^ ^]^ Flattening and collapse of regenerated tissue were observed in scaffolds without an nHA gradient.^[^ ^102^ ^]^ The extensive nHA at the bottom of gradient scaffold fully exerts the role of osteoconduction and osseointegration, and the well‐regenerated subchondral bone maintains the biomechanical properties of entire osteochondral region, thereby providing a good platform for cartilage repair tissue.

In addition to β‐TCP and nHA, several other minerals exhibit the potential to repair osteochondral defects when they are developed to be gradient scaffolds. Typically, the gradient scaffold formed by mixing Mn^2+^ and bioglass into hydrogels with different crosslinking strengths simultaneously facilitated osteochondral repair.^[^ ^109^ ^]^ The nanofiber plates incubated in simulated body fluids for different amounts of time absorbed different amounts of minerals. The A‐MSCs exhibited a gradient osteoblast phenotype in consistence with the mineral gradients.^[^ ^110^ ^]^ The osteoinductive properties of mineral and scaffold's matrix hardness and surface roughness exerted an improved osteogenic effect with the increased mineral content.

#### 2.4.3 Osteochondral scaffolds with other gradient osteochondrogenesis‐inducing factors

In addition to growth factors, some substances in the differentiation induction medium also promote differentiation induction. Insulin has been shown to promote chondrocyte proliferation and ECM deposition and induce osteogenesis.^[^ ^111^ ^]^ β‐GP is an essential component of the osteogenic medium that produces the phosphate needed by nHA and is used as an intracellular signaling molecule to regulate the expression of various osteogenic genes, such as *OPN* and *BMP‐2*.^[^ ^112^ ^]^ PCL scaffolds containing continuous gradient levels of insulin and β‐GP influenced cell structural development and mineralization. More Col deposition and cartilage‐like cell formation occurred as the insulin concentration increased. In contrast, the degree of mineralization increased with the rising β‐GP concentration.^[^ ^113^ ^]^ BM‐MSCs were effectively induced to differentiate into calcified cartilage when the chondrogenic and osteogenic media were mixed at 1:1 (*V*/*V*). Therefore, Jin et al. cocultured BM‐MSCs with fibrous mesh sheets in different mixed proportions of chondrogenic and osteogenic media.^[^ ^114^ ^]^ The gradient sheet structures implanted in the osteochondral defects of rabbit knee joints promoted tissue regeneration and produced an intact osteochondral layer.

Taurodeoxycholic acid (TUDCA) is a bile acid that plays a role in angiogenesis and inhibits the adipogenic differentiation of MSCs. Low concentrations of TUDCA (25 μm) promoted the osteogenic differentiation of MSCs, whereas high concentrations of TUDCA (2500 μm) enhanced the cell proliferation and chondrogenesis of MSCs.^[^ ^115^ ^]^ Scaffolds with a TUDCA gradient recruited BM‐MSCs to the osteochondral defect and induced chondrogenesis and osteogenesis.

Signals carried by the inducing factors are received by MSCs and regulate the expression of downstream genes, thus affecting MSC differentiation. When the inducing factors are distributed in a gradient, the differentiation of tissues with corresponding functions will be induced in a specific space.

## 3 CONCLUSIONS AND FUTURE DIRECTIONS

This review summarizes the gradient scaffolds for osteochondral tissue engineering. Emerging studies have made remarkable progress in repairing and regenerating osteochondral tissue in vitro and in vivo. Nevertheless, the long‐term repair effects are not satisfactory, which mainly can be attributed to the ignorance of gradient structure of osteochondral tissue, resulting in the lack of accurate osteochondral biomimetics. The regenerated cartilage and subchondral bone were poorly integrated, and the new tissue was not anchored firmly to the surrounding normal tissue. With the development of gradient scaffolds and increasingly precise biomimicry, we are eager to improve the treatment efficacies of osteochondral injuries. Conclusively, advanced osteochondral scaffolds still require further investigation for the clinical translation of these biomaterials.

Under multiple factors, endogenous and exogenous stem cells differentiate along different lineages and form functional tissues. Cartilage has been relatively hypoxic for a long time, as natural cartilage lacks vascular and lymphatic structures, and nutrients mainly derive from the infiltration of synovial fluid. The reduced pore sizes and lower porosity decrease the permeability of substances and keep stem cells in a relatively nutrient‐deprived state, thereby inducing the chondrogenic differentiation of stem cells. In contrast, in fracture repair, excessive incisions in the soft tissues surrounding the bone disrupt the supply of blood to the bone, leading to delayed union or non‐union of fracture. Therefore, osteogenic differentiation requires a relatively abundant nutrient supply, which can be provided by scaffolds with larger pore sizes and higher porosity.

Pores are critical factors for cell growth, as they provide the cell with a survival microenvironment. Interconnected 3D porous structures facilitate cell migration and adhesion into materials and transport nutrients and metabolites. For osteochondral tissue engineering, a scaffold with smaller pore size, lower porosity, and square shape is required for chondrogenic differentiation, while osteogenic differentiation requires the opposite. Thus, scaffolds with gradient pores will likely be used in the complex structures for osteochondral repair. Moreover, calcified cartilage structures are present during the change from cartilage to the subchondral bone, so instead of simply increasing the pore size, a dense scaffold layer should be added at the appropriate location to mimic the calcified cartilage layer. Among the various techniques for synthesizing osteochondral scaffolds, 3D printing technology has many advantages, such as its ability to control the required pore size and shape to synthesize interconnected 3D porous scaffolds for a smooth transition between regions.^[^ ^116^ ^]^ Future studies should focus on more accurately designing the pore gradients of scaffolds to optimize the osteochondral repair effect, in‐depth explorations of the mechanisms by which pores affect stem cell differentiation, and continuously improving synthetic technology to develop methods that are economical, effective, convenient, and easily translated into clinical practice.

The physical properties of scaffold materials, such as mechanical strength, viscoelasticity, stiffness, and surface roughness, also influence the differentiation of stem cells. Gradient changes in mechanical strength from cartilage to the subchondral bone layer can be achieved by selecting specific materials or changing the content of each component. However, the porosity of scaffold is also an essential factor affecting the mechanical strength. The regeneration and repair of subchondral bone require a large porosity to support material permeability and high mechanical strength to support neocartilage. Future studies will focus on balancing the porosities and mechanical strength of scaffolds and maximizing the synergistic effects of two factors in osteochondral repair.

Viscoelasticity, stiffness, and surface roughness mainly affect cell proliferation, migration, and differentiation. The stem cells tend to differentiate into specific phenotypes in the microenvironments whose stiffness is comparable to that of corresponding tissue. The matrix stiffness would affect the local adhesive structures and cytoskeleton. When cells adhere to a matrix, they sense the pulling forces as the matrix deforms, which generates the corresponding cell signals to induce differentiation. The surface roughness of materials affects the anchorage of cells. Rough materials benefit to the adhesion between cells and materials, making cells prone to deformation and elongation during migration. The expanded shape favors the osteogenic differentiation of stem cells. In summary, the characteristics of materials are important factors that influence the directed differentiation of stem cells, and the mechanisms underlying their effects on stem cell differentiation are worthy of in‐depth study. In future studies, it is essential to establish gradients of these material characteristics to control the differentiation of stem cells in different layers of scaffolds and precisely regenerate tissues resembling the natural osteochondral structure. Noteworthily, researchers should focus on the dynamics of materials during the repair process. The scaffold matrix provides mechanical support for tissue regeneration and dynamically regulates cell proliferation and differentiation.^[^ ^117^ ^]^ When stem cells are initially colonized in the scaffold, the soft matrix promotes stem cell proliferation and increases the efficiency of stem cell differentiation. As the repair proceeds, changes in the matrix affect the direction of stem cell differentiation.^[^ ^118^ ^]^ Hence, designing materials that dynamically adapt to the tissue repair process is another way to improve scaffold biomimicry.

In addition to the effect of scaffolds, inducing factors in tissue engineering also play essential roles in the directed differentiation of stem cells. They bind to specific receptors on the cell surface or in the cell and activate downstream signaling molecules in the nucleus, further affecting the transcription and expression of specific genes. The mechanisms of stem cell differentiation in response to TGF‐β and BMP are relatively straightforward. Studies have shown that establishing the reverse gradient of TGF‐β and BMP is conducive to osteochondral tissue regeneration, like promoting specific cell differentiation and producing the corresponding ECMs. Nevertheless, the effect of long‐term repair is unsatisfactory due to the imperfect release strategy. In addition, cartilage is a hierarchical structure, and the GAG and Col contents in the cartilage ECMs are highest in the middle layer. However, in some studies, the gradient of growth factors is only monodirectional, increasing or decreasing, which is unsuitable for cartilage regeneration. Motivated by this rationale, we should further investigate how to accurately control the content and release of loaded growth factors to regenerate cartilage tissue with normal physiological function and focus on how to induce the production of calcified cartilage at specific locations.

Similarly, as an essential part of the bone, minerals are widely used in gradient scaffolds to induce the osteogenic differentiation of stem cells. Moreover, scaffolds with mineral gradients showed the ability to promote the early repair of subchondral bone and the formation of calcified cartilage. Another interesting point is that adding minerals always enhances the mechanical strength of materials, which is also beneficial to the growth of subchondral bone. However, adding minerals may change the surface or internal structures of scaffolds, affecting their degradation or the release of other substances from the scaffold. Therefore, characterizing how minerals affect the performances of scaffolds is a problem to be addressed. In addition to growth factors and minerals, many other induction factors are loaded onto gradient scaffolds, which exhibit a certain degree of osteochondral repair. It is also essential to develop more optimal osteochondral‐inducing factors. Nucleic acid drugs have been widely used for tissue engineering because of their tissue infiltration ability, stable structure, and flexible programming.^[^ ^119^, ^120^, ^121^ ^]^ Establishing gradients of these inducing factors is also a future research focus.

Degradation products of materials also affect stem cell differentiation,^[^ ^122^ ^]^ and if these degradation products can be thoroughly investigated and utilized in place of the function of inducing factors, then it would significantly facilitate the clinical translation of osteochondral gradient scaffolds. Moreover, the complex inflammatory response experienced in the early stages of osteochondral injury cannot be ignored, and excessive inflammation makes osteochondral repair slow.^[^ ^123^, ^124^ ^]^ The scaffold and controlled release of factors must adapt to changes in the microenvironments of osteochondral regeneration in the temporal dimension.^[^ ^125^ ^]^ Scaffolds that exert initial anti‐inflammatory and long‐term repair effects demonstrated better osteochondral repair.^[^ ^126^ ^]^

In conclusion, gradient scaffold is a valid strategy for repairing osteochondral defects, but further research is needed to achieve clinical translation. Future research should focus on (1) elucidating the mechanism and signaling pathways that influence the physical properties (pore, mechanical, micromorphology, and so forth) of scaffolds for stem cell differentiation; (2) investigating the effect of chemical structure of the materials or their degradation products on stem cell differentiation; (3) designing materials that are dynamically adapted to the tissue repair process; (4) searching the optimized induction factor and then accurately controlling the loading amount and long‐term stable release of induction factors; (5) taking the calcified cartilage layer into consideration; and (6) evaluating the long‐term effect of osteochondral repair.

## AUTHOR CONTRIBUTIONS

Yachen Peng and Yaling Zhuang contributed equally to this work. Yachen Peng: Conceptualization, Investigation, Resources, Writing—Original Draft, Writing—Review & Editing. Yaling Zhuang: Writing—Review & Editing. Yang Liu: Writing—Review & Editing. Hanxiang Le: Writing—Review & Editing. Di Li: Writing—Review & Editing. Mingran Zhang: Writing—Review & Editing. Kai Liu: Writing—Review & Editing. Yanbo Zhang: Conceptualization, Writing—Review & Editing, Supervision, Project administration, Funding acquisition. Jianlin Zuo: Writing—Review & Editing, Supervision. Project administration, Funding acquisition. Jianxun Ding: Conceptualization, Writing—Review & Editing, Supervision, Project administration, Funding acquisition.

## CONFLICT OF INTEREST STATEMENT

The authors declare no conflict of interest.
